# Strategies and Supports to Improve Retention of Personal Care Workers in Residential Aged Care: Insights From a Qualitative Study

**DOI:** 10.1177/01640275251383540

**Published:** 2025-10-08

**Authors:** Britt O’Keefe, Eva Yuen, Susan Perlen, Alison M. Hutchinson

**Affiliations:** 1 School of Nursing and Midwifery, Centre for Quality and Patient Safety Research (QPS) in the Institute for Health Transformation, 2104 Deakin University, Geelong, VIC, Australia; 2 Monash Health, Clayton, VIC, Australia; 3 Barwon Health, Geelong, VIC, Australia; 4 Nursing, Faculty of Health Sciences, University of Southern, Denmark; 5 Honorary Fellow, National Ageing Research Institute, Parkville, VIC, Australia

**Keywords:** aged care workers, attraction, human resource management, intention to stay, nursing home

## Abstract

To identify strategies and supports to enhance retention and reduce turnover of personal care workers in residential aged care. Interviews were conducted from August 2023 to September 2024 with 20 former personal care workers who had worked in residential aged care. Data were analyzed using reflexive thematic analysis. Five major themes were identified: (1) managers who engage with residents, (2) managers who value, recognize and support personal support workers, (3) comprehensive onboarding and continuing training programs, (4) strengthening the workforce and working arrangements, and (5) inclusive, collaborative and respectful work culture. Persistent workforce shortages in residential aged care underscore the need for targeted strategies and supports to retain staff. The findings of this study illustrate strategies and supports to promote personal care worker retention, offering practical guidance for further consideration by residential aged care operators, policymakers, and researchers.

## Introduction

Personal care workers (PCW; also known as certified nursing assistants, nursing assistants and personal care aides) are widely regarded as the ‘backbone’ of the residential aged care (RAC), direct care workforce (Wang et al., 2024). Given that they spend the most amount of time with residents, PCWs play a pivotal role in improving residents’ quality of life in RAC, by assisting with the provision of round-the-clock care (Gibson et al., 2023), along with supporting activities of daily living such as assistance with eating, bathing and dressing (Wang et al., 2024). Responsibilities of PCWs extend beyond physical assistance to include emotional, spiritual and psychosocial support, contributing to the overall wellbeing and quality of life of residents in aged care.

The global population of individuals aged 80 years and over is projected to triple, from 143 million in 2019 to 429 million by 2050 (World Health Organization, 2024). This demographic shift, compounded by declining fertility rates, smaller family sizes, and increased female workforce participation, is expected to drive growing demand for aged care services, particularly in RAC (Cullen, 2019). It is estimated that nearly two-thirds of older people will require RAC services and support during their lifetime (Harris-Kojetin et al., 2016), highlighting the critical need for a sustainable direct care workforce.

Across high-income settings, most notably Australia, the United Kingdom (UK), and the United States of America (USA), the term residential aged care (RAC) facility, is used interchangeably with terms such as nursing home, long-term care facility, or care home. These systems vary widely by country in terms of funding models, regulatory oversight, governance structures, and staffing requirements (Dyer et al., 2019). Despite these differences, many countries are shifting toward a person-centered care approach in underpinning policies and directing practices that address the uniqueness of each RAC recipient (Wilberforce et al., 2017).

In RAC, direct care workers, including registered nurses (RN), enrolled nurses (EN), PCWs and allied health professionals, provide care as a core component of their roles (Australia Government Department of Health (AGDH), 2020). Over the past two decades, Australia’s aged care workforce census has reported a substantial shift in workforce composition, characterized by a marked increase in the proportion of PCWs and a corresponding decline in the number of full-time equivalent RNs and ENs (AGDH, 2020). Between 2003 and 2020, the proportion of PCWs employed in RAC across Australia increased from 56.5% to 72.1% (AGDH, 2020). Consequently, RAC operators in Australia have become increasingly reliant on PCWs to provide essential care and support to residents.

In the aged care sector, employee turnover has been extensively studied, whereas employee retention has received limited attention (Rosen et al., 2011). Employee retention refers to the continual employment of individuals within the same organization for a defined period (Castle, 2021), while employee turnover refers to employees’ departure from an organization, transfer to other areas of the labor market, or an occupation change within the same organization (Hayes et al., 2012). Although employee retention and employee turnover are often regarded as inverse concepts, with a reduction in turnover assumed to reflect retention, research suggests that the factors influencing employees’ decisions to remain in their roles (retention) may differ from those driving their decisions to leave (Nancarrow et al., 2014).

Few studies have examined the factors influencing both employee turnover and retention among the PCW workforce within a single study. In a qualitative study of 47 PCWs, Mittal et al. (2009) compared ‘stayers’ (those with three or more years in the same job) and ‘leavers’ (those who had changed jobs in the past three years) and found that the drivers of turnover were different from the drivers of retention. Turnover was associated with a lack of respect from management for the work, poor management, work-family imbalance, difficulty of work tasks, and alternative employment opportunities, whereas retention was linked to a sense of calling, resident advocacy, personal relationship with residents and families, spirituality and job flexibility (Mittal et al., 2009). A longitudinal study of 620 PCWs, comprising stayers, switchers (those who had changed jobs once or twice the past three years), and leavers, that treated turnover and retention as reciprocal outcomes revealed that turnover was significantly positively predicted by high turnover intentions, while retention was significantly supported by high job satisfaction, which was negatively associated with turnover (Rosen et al., 2011). In a secondary analysis of the USA 2004 National Nursing Home Survey data, Donoghue (2010) found that only organizational characteristics, such as not-for-profit and higher average occupancy levels, were significantly associated with increased retention among PCWs (Donoghue, 2010). Similarly, following secondary analysis of four data sets, Kennedy et al. (2020) reported that for-profit ownership was the only variable significantly associated with both high turnover and low retention among PCWs.

Research has revealed the impact of PCW retention on the quality of care provided to older people in RAC (Kimmey & Stearns, 2015). Continuity of care, wherein PCWs consistently care for the same residents, has been shown to enhance resident well-being by facilitating familiarity, accommodating individual preferences, and enabling the early identification of health concerns (Morgan et al., 2013). For PCWs, continuity of care also offers intrinsic rewards, including greater job satisfaction and professional fulfilment (Boun et al., 2023).

Little attention has been paid to exploring the experiences and perspectives of former PCWs, who are uniquely positioned to provide insights into unmet needs, workplace challenges, and barriers to retaining PCWs (Kennedy et al., 2020; Rosen et al., 2011). Exploring retention strategies through this lens provides a unique opportunity for this underrepresented group (Rosen et al., 2011) to reflect on their experiences and provide their perspectives on how RAC operators could improve their workforce models and supports to promote employee retention. Given the increasing reliance on PCWs and the global aging population, understanding how to retain this workforce is critical. Therefore, the study aim was to explore the strategies and supports to enhance PCW retention and reduce turnover from the perspectives of former PCWs in RAC.

## Methods

### Study Design

An exploratory, descriptive qualitative design was used in this study to explore participants’ perspectives and experiences. This methodology is particularly useful when the topic remains under investigation, is under-researched, and sufficient empirical understanding is lacking (Hunter et al., 2019). In preparing this manuscript, the 32-item Consolidated Criteria for Reporting Qualitative research (COREQ) Checklist was used (Tong et al., 2007).

### Ethical Considerations

The study received ethics approval (reference number: XX ID 2022-154). Prior to each participant’s scheduled interview, written informed consent was obtained. All interview data were securely stored in accordance with institutional research conduct and data management policies, as well as the Australian Code for the Responsible Conduct of Research (National Health and Medical Research Council, 2018).

### Setting and Participants

Purposive sampling was selected to ensure inclusion of information-rich cases and to yield in-depth insights into the phenomenon under investigation, rather than aiming for empirical generalizations (Hennink et al., 2020). Individuals were eligible for inclusion if they were aged 18+ years, had previously worked as a PCW in a RAC facility, had voluntarily left the aged care sector within the last five years, could read and speak English, and were able to provide informed consent.

Participants were recruited through Facebook. Interested individuals were invited to complete an online pre-screening questionnaire to assess their eligibility. Eligible individuals were invited by email to a non-recorded, informal pre-interview meeting, during which the study’s purpose was discussed, inclusion criteria confirmed, and requirements for informed consent were addressed. If the individual was willing to proceed, an interview time was scheduled based on their availability. Prior to the interview, participants were emailed a Plain Language Statement Consent Form and confirmation of their scheduled interview time. Written consent was recorded with an electronic signature, and verbal consent was audio recorded at the commencement of the interview. Interviews were conducted between August 2023 and September 2024. We aimed for a sample of approximately 20 individuals to capture a rich, diverse and relevant range of views (Hennink et al., 2020) and to achieve information power (Malterud et al., 2016).

### Data Collection

A semi-structured interview guide was developed, with open-ended questions to encourage conversation on employee retention, and with additional questions to expand on participant responses. Examples of questions used during the interviews are “what did your organization do (if anything) to encourage personal care workers to stay?” and “what advice would you give to your previous manager to make sure that personal care workers want to stay working in aged care? The interview guide was pre-tested with two PCWs from the author’s professional network to ensure clarity, coherence, structure and alignment of the interview questions. All interviews were conducted online using the Zoom teleconferencing platform, with both audio and video recording to ensure comprehensive data capture. Additionally, Zoom’s built-in chat-to-text function was also utilized to collect supplementary text-based data.

### Data Analysis

To ensure transcription accuracy, Zoom audio recordings were cross-referenced with the automated text-based transcripts, reviewed and transcribed verbatim. Identifiable information, including names and workplaces, was redacted for confidentiality and quotes were revised for clarity and readability (Barbour, 2014). Data analysis was guided by Braun and Clarke’s (2006) six-phase reflexive thematic analysis process. This approach was selected to produce codes that accurately reflect the content and meaning of participants’ data (Braun & Clarke, 2006). Each transcript was read multiple times by the first author to develop an initial understanding of the data. A descriptive, inductive coding approach was used to capture explicit meanings within each transcript. Using an iterative process, initial codes, along with corresponding participant quotes, were transferred into a Microsoft Excel spreadsheet by the first author, who also undertook the initial analysis. Codes and participant quotes were subsequently reviewed by all authors, refined, combined or deleted to gain deeper insight into the data. Meaningful clusters of codes were then grouped by the first author to identify patterns of meaning, resulting in the generation of initial subthemes and themes. Themes and subthemes were iteratively refined by all authors, with consensus on the final set achieved through collaborative review and discussion.

### Trustworthiness

Trustworthiness, or methodological rigor, was maintained in this study by addressing Lincoln and Guba’s (1985) criteria: credibility, confirmability, transferability, and dependability. In this study credibility was supported through an inductive analytic process that minimized bias, with participant language closely informing subtheme construction. Participants were also invited to review their de-identified transcripts to enhance transparency and interpretive depth. Confirmability was established through the reflexive journaling, audit trails, and systematic data management to demonstrate traceability of analytic decisions. Transferability was addressed through providing thick descriptions supported by illustrative quotes. Finally, dependability was ensured through systematic data management, maintenance of audit trails, and regular team discussions to support theme development.

## Findings

### Characteristics of Participants

Twenty former PCWs participated in interviews that ranged in duration from 30 to 169 minutes (average: 82 minutes). Participants were aged between 21 and 70 years (mean: 49.4), and most were female (95%). Over half of the sample (12) were born in Australia (60%), with three born in England, two in China and one each in the United States, Hong Kong, and the Philippines. Years of experience working as a PCW ranged from 1.5 to 40 years (mean: 8.62 years). The age and gender of the participants in this study are consistent with demographic characteristics of the broader Australian RAC PCW workforce (Australian Government Department of Health, 2020). Almost all participants (90%) expressed a desire to return to their previous roles in RAC if appropriate retention strategies and supports were implemented.

### Thematic Findings

Analysis of the interview data revealed five major themes and 10 subthemes that captured strategies and supports to promote PCWS retention: (1) managers who engage with residents, (2) managers who value, recognize and support PCWs’, (3) comprehensive onboarding and continuing training programs, (4) strengthening the workforce and working arrangements, and (5) inclusive, collaborative and respectful work culture. The themes, subthemes and illustrative quotes are presented in Table 1.

**Table 1:** Participant Perceptions of Strategies and Supports Required to Promote Retention of Personal Care Workers: Themes, Subthemes and Supporting Quotes

Theme	Subtheme	Supporting quotes
1. Managers who engage with residents		“Just acknowledge [residents] as individuals and get to know their names. The main…manager…he'd come into the room and walk straight past all of the residents. He wouldn't even know one name in that whole room. [residents] used to say, here he comes, doesn't even know who we are…I just thought, yeah, I mean, get to know the resident’s names” [P_009] “[Management] actually engaging with [residents] directly face to face as well…I'm not the conduit to them, [management] are actually coming in and speaking and engaging with the [residents] themselves” [P_014] “The previous manager, every morning without fail, before she went anywhere near the office…she would go around every [resident’s] room and say, good morning, and that's what it should be, you know. [Ask the residents] ‘have you any problems, you alright, you know, you're right’, do that don't sit in your office, be a part of [residents] lives” [P_018]
2. Managers who value, recognize and support PCW’s	Employee recognition from managers	“…[management will] get the best out of people if they acknowledge the way [personal care workers] work and what they have done…if somebody [personal care workers] had done something really nice… Just a little bit of acknowledgement [from management] for a job well done” [P_009] “[Management] used to give us …nothing much like a $10 voucher like a grocery voucher or something like that which is kind of good. Now we don’t receive anything…you work here for years, they don't acknowledge us, the hard work that we do. However, they do acknowledge the RNs, which is…a bit a downfall…it's like a kick in the guts…like the [personal care worker] did all the hard work…[P_008] “Try and keep the staff morale up, now and then they do like a little food thing and keep, sort of, doing that for lunchtime” [P_001] “You give gift cards like a $50 voucher for doing an extra shift” [P_005]
	Visible, supportive, and approachable managers	“[Management] communicating directly [with care workers] and…not locked up in head office, they're actually out visiting the facilities on a regular basis**…**[and when short staffed] even the management come along and also assist as well…so, many hands make light work” [P_014] “We've got to have more support, when we're short [staffed] it's all hands on the floor to help everybody” [P_019] “If you are short staffed, you know, [have managers] come around and see what [they] can do…even if [management] just come around and feed someone, you know…show more interest, be on the floor and be approachable” [P_004]
	Managers who value PCWs’ knowledge of residents to enhance care	“[Information about the resident] was never passed on because it wasn't as per her care plan. There was so much that you get to know the person as a [personal care worker], because you're interacting with them daily, that doesn't get passed on, you know, that past trauma and the reasons for their behaviors and the stuff that would make it significantly better, just doesn't happen” [P_011] “We are the closest ones working with the residents, you need to listen to what we were saying too, about [the residents]” [P_002] “[Management] need to listen to their care workers more, and that we're not making suggestions just because we want to make them, we're making them because it's going to not only improve the business but improve the resident's quality of life at the end of the day” [P_010]
3. Comprehensive onboarding and continuing training programs	Onboarding and structured buddy program	“Have a good orientation and buddy shift system, because when [care workers] have a good buddy shift [when they start the job] I think they can take more knowledge in. So, it can help them to make them feel like, oh, this is how to do it” [P_013] “I got two buddy shifts and one of them, I can remember, was with somebody who didn't want to have to do buddy shifts...it was like they were resentful of having somebody having to follow them around and work with them...if you've got buddy shifts, you have got to be people who want to …people who volunteered [to be buddy rather than being tasked]” [P_020]
	Continuing training and skill development	“I think it's good that they… keep doing staff training. I think that's essential, because everything does change continually” [P_001] “I think more training needs to be done, constantly to make sure your knowledge and experience are up to date” [P_015] “I had no training at any point other than I did Certificate 3 [formal qualification], which was done once I had already started working…I had no training in palliative care at all at any stage of my 17 years…I've never had any dementia training either…any sort of well-being training” [P_012]
4. Strengthening the workforce and working arrangements	Strengthening workforce skill-mix and staffing levels	“…because the nurse on shift has a huge influence on how the shift goes and what support [personal care workers] get and [learn]. You can learn heaps and stuff. So, retaining the good nurses makes a huge, positive impact, for I think retaining care staff” [P_020] “…more staffing, more qualified staffing that know what they're doing…[who are] going to stay in that position for a long period of time” [P_003] “I think give them more hours…if they, offer [care workers] like full time, cause rarely they give personal care workers full time positions” [P_008]
	Flexible working arrangements and supports	“We need to accept volunteers to come in and help out where possible” [P_019] “…[RAC operators] being very flexible, like we had some [personal care workers], you know, can't come in at certain time, they were very flexible like that” [P0_008] “The agency tried to be as flexible as possible, and you could say, no, to any shift, you don't have to, you're not committed to any shift” [P_020] “Somebody came in for a short shift every day… that really did, I think, help the workload” [P_009] “Having somebody HR available once a week, to be answering questions [from personal care workers] till 8 o'clock at night or something for an hour” [P_020]
	Prioritizing employee health, mental well-being and injury recovery	“I think, you need to know that it's okay to look after yourself…there needs to be more support for staff, on the coal face in any capacity, in terms of supervision and access to professional counselling” [P_007] “Some of them have their little programs, they bring in things like fitness gyms to the organization” [P_014] “We need to have a limit on how many hours someone can work in a week” [P_019] “You can't really rush [injured staff to return to work]….I did hear stories like someone's injured, they rest, they have two days off, and then management just try to rush them back to work, and they get worse, and it just doesn't give them enough time for them to heal…Just really look after the people who are working for you and when they get injured…help them with the workers compensation process and also getting them well” [P_005]
5. Inclusive, collaborative, and respectful work culture	Inclusive and open communication in decision-making	“…nothing hidden, no hidden agendas or anything like that, it's an open book… actively encourage open communication with no reprisals or anything like that” [P_014] “…All of us irrespective of who we are [e.g., personal care worker], where we are [in the organization hierarchy], we want to feel like we're part. We want to be collaborators, co-operators. We want to collaborate, cooperate co-own the process. We want to be part of something, and not just lip service, it has to be real” [P_007] “It's just that, not being asked or considered is the biggest thing, we can, you know, brainstorm together come up with how we can manage [communication and feedback] and just feel like we're, we're all part of this machine and not the people on the bottom” [P_016]
	Respectful, equitable and inclusive workplace	“I just think that if you are the personal care worker, you want to be treated with respect” [P_013] “Be kinder to how you [management] speak to people” [P_002] “More respect given to the people that do that job, because people say, you're an aged care worker, but you're not just an aged care worker” [P_009] “Being fair across the board, not just newbies, not just your favorite shift or your favorite people, but they treat everybody with the same respect and the same standards, making sure that the same set of rules applies to everybody” [P_020] “We need to work as a team, and we need to support each other” [P_019]

### Theme 1: Managers Who Engage with Residents

Participants valued managers who engaged and demonstrated a personal connection with residents. Managers who engaged in regular and meaningful interactions with residents, such as greeting each person by name and engaging in daily conversations, were perceived as demonstrating that they ‘cared’ for the residents as people. This contrasted with managers who were disengaged and perceived as distant to the residents. This was displayed through their lack of acknowledgement of residents as individuals, which PCWs reported was noticed by residents and negatively impacted employee morale.

### Theme 2: Managers Who Value, Recognize and Support PCWs

Three sub-themes related to employee recognition and supportive leadership: (1) employee recognition from managers, (2) visible, supportive, and approachable managers, and (3) managers who value PCWs’ knowledge of residents to enhance care.

#### Employee Recognition

Participants emphasized the importance of recognition from managers to enhance employee retention. Several participants described the value of their work contributions being acknowledged by managers and the positive impact on staff morale. One participant reported being frustrated when recognition was disproportionately directed towards registered nurses, leaving PCWs feeling overlooked and undervalued. While some viewed small, informal gestures of appreciation, such as providing lunch to staff, as meaningful methods to recognize PCWs’ contributions, others perceived these small gestures as insufficient to demonstrate genuine appreciation for the work.

Recognition was also described in both financial and non-financial terms. One participant recommended using financial rewards, such as additional compensation for accepting extra shifts, to maintain employee morale. Other participants discussed the importance of non-financial rewards, such as social activities (e.g., Christmas parties and complimentary meals) to foster PCW engagement, boost morale, and retain staff.

#### Visible, Supportive, and Approachable Managers

Participants highlighted the importance of visible, supportive, and engaged leadership in retaining PCWs. They advocated active leadership, emphasizing that managerial visibility was demonstrated through regular presence across the care facility rather than being confined to offices. Managers who sought to meaningfully engage with PCWs and other staff during shifts, such as engaging in conversation, also fostered a perception of managerial visibility among PCWs. Concerns were raised regarding the absence of managers and limited leadership presence, with some staff being unaware of changes in managerial appointments.

Beyond visibility, having supportive and responsive leadership was considered essential for retaining PCWs. Multiple participants discussed the value of hands-on managerial support, particularly during staff shortages. Personal care workers valued managers who alleviate workload pressures by assisting with time-intensive tasks, such as feeding residents. Additionally, participants wanted managers who were approachable and who demonstrated genuine interest in PCWs’ lives, to foster a more positive and cohesive work environment.

#### Managers Who Value PCWs’ Knowledge of Residents to Enhance Care

Participants underscored the need to be heard and to have their knowledge of residents recognized and valued by managers. While participants described themselves as having the closest relationship with residents, they expressed frustration over limited opportunities to share and contribute their insights through formal mechanisms, such as notes in care plans, to enhance resident care. The lack of acknowledgement from managers of PCWs’ experiential knowledge and PCWs’ inability to openly share information were perceived as undermining their value, hindering care quality, and a barrier to retaining PCWs in their role.

### Theme 3: Comprehensive Onboarding and Continuing Training Programs

Two sub-themes emerged related to comprehensive onboarding and continuing training programs: (1) onboarding and structured buddy program, and (2) continuing training and skill development.

#### Onboarding and Structured Buddy Program

A comprehensive onboarding program, including formalized buddy shifts, was identified as an effective strategy to support PCWs at the commencement of their employment to promote employee retention. Participants emphasized that a structured onboarding process incorporating peer support helped new staff become oriented to the demands of their role. Additionally, participants stressed the need for a structured buddy program and highlighted the importance of carefully selecting ‘buddies’ to effectively support new staff to strengthen workforce retention.

#### Continuing Training and Skill Development

Participants emphasized the importance of continuing training to enhance job readiness and competency for PCWs to promote employee retention. Continuing training was seen as essential for maintaining staff competencies, ensuring up-to-date knowledge, and meeting the evolving requirements of the PCW role. However, some participants reported a lack of additional training beyond completing a Certificate 3 (formal qualification), while others identified knowledge gaps in specialized areas essential to high-quality care, particularly for palliative and dementia care.

### Theme 4: Strengthening the Workforce and Workings Arrangements

Three sub-themes emerged related to the importance of strengthening the workforce and working arrangements: (1) strengthening workforce skill-mix and staffing levels, (2) flexible working arrangements and supports, and (3) prioritizing employee health, mental well-being and injury recovery.

#### Strengthening Workforce Skill-Mix and Staffing Levels

Participants highlighted the importance of workforce skill-mix and adequate staffing levels in enhancing employee retention. Retaining nurses was described as integral to the provision of clinical oversight, guidance and support, enabling PCWs to effectively manage the complex needs of residents. Participants discussed the need for additional staff to alleviate workload pressures for PCWs and more qualified staff to improve the quality of care and reduce the risk of burnout among PCWs. Additionally, strategies such as expanding to full-time positions to promote job security and utilizing volunteers were suggested to increase retention among PCWs.

#### Flexible Working Arrangements and Supports

Having flexible work arrangements and supports, such as enabling PCWs to choose shifts that aligned with their personal schedules, was described as contributing to staff retention. One participant highlighted the advantage of having greater shift flexibility when employed through an agency rather than being directly employed by the RAC. Implementing shorter shifts as a flexible working arrangement was proposed to alleviate workload pressures and enhance staff retention. Providing more formal support for PCWs, such as extending human resource services beyond standard business hours to accommodate the needs of PCWs working afternoon and evening shifts, was perceived to foster a supportive and responsive work environment.

#### Prioritizing Employee Health, Mental Well-Being and Injury Recovery

Supporting employee physical and mental health was identified as a strategy to promote employee retention. Participants discussed the importance of self-care awareness and the need for workplace well-being programs and introducing limits on the number of hours PCWs can work in a week to prevent employee burnout and fatigue. Access to professional support services, including counselling, was also highlighted as a way to address the emotional and psychological demands inherent in the PCW role and reduce workplace stress.

Comprehensive support for injured workers, comprising a compassionate and structured return-to-work program, was identified as facilitating employee retention. One participant discussed the importance of ensuring proper assistance, from the initial claims process through to a well-supported and structured transition back to work, to prioritize employee well-being and motivate PCWs to return and remain in their role.

### Theme 5: Inclusive, Collaborative and Respectful Work Culture

Two sub-themes related to the importance of an inclusive, collaborative and respectful work culture: (1) inclusive and open communication in decision-making, and (2) respectful, equitable and inclusive workplace.

#### Inclusive and Open Communication in Decision-Making

Participants discussed the importance of inclusive, open and respectful communication between PCWs and management to foster a positive workplace culture and encourage staff retention. Feeling heard and included in decision-making processes was identified as empowering PCWs in their role. Participants sought to be seen as collaborators and co-operators in the organization, and for their input and feedback on care provision to be heard by senior staff; key steps in enabling PCWs to feel included and valued in the workplace and to foster a positive and inclusive workplace culture.

#### Respectful, Equitable and Inclusive Workplace

A respectful, equitable and inclusive workplace was also identified as an important factor in supporting staff retention. Participants described the value of being treated with respect and highlighted the role managers play in demonstrating kindness, which fosters job satisfaction and reinforces workforce commitment. Equitable workplace treatment was seen to create a supportive work culture, and participants wanted consistent and impartial managerial practices to ensure uniform expectations for all staff. Additionally, participants discussed the importance of an inclusive workplace that fosters teamwork and mutual support.

## Discussion

Employee retention remains a significant challenge for many RAC operators, yet it has received comparatively less attention in existing literature than employee turnover. To the authors’ knowledge, this is one of the few studies to focus specifically on the perspectives of former PCWs who have left the aged care sector, to identify strategies and supports that could enhance retention among PCWs in RAC. By focusing on former rather than current workers, this study identified the key factors that might have encouraged them to stay. These retrospective accounts, contribute to a relatively scarce body of research, provide candid and distinctive insights for RAC operators and offer a robust evidence base to inform more targeted retention strategies. Our findings revealed five major themes: (1) managers who engage with residents, (2) managers who value, recognize and support PCWs, (3) comprehensive onboarding and continuing training programs, (4) strengthening the workforce and working arrangements, and (5) inclusive, collaborative and respectful work culture.

Building on existing literature and considering the global shift toward person-centered care (Teisberg et al., 2020), our findings showed the importance of managers engaging with residents. The importance of strong, meaningful relationships between residents and managers was key to enhancing employee retention. The findings from this study align with Hollinger-Smith’s (2003) three Rs of retention: relationships, respect and recognition, emphasizing their critical role in retaining employees. Participants in this study emphasized the importance of managers developing relationships not only with PCWs but also with residents, reinforcing the value of interpersonal connections in retention efforts. Although our findings echoed those of Thwaites et al. (2023), whose recent systematic review of 49 quantitative, qualitative, and mixed-methods studies identified positive relationships with peers, colleagues, supervisors, management, residents and families as key drivers of aged care worker retention across 17 studies, their review examined both recruitment and retention across a broad cohort of direct care workers. In contrast, our qualitative study offers a distinct contribution by focusing exclusively on PCWs who had left the aged care sector. This focus enabled an in-depth exploration of the factors that would have promoted retention, rather than relying on intention to stay as a proxy, as in Thwaites et al.’s (2023) review. As such, our study complements and extends the work of Thwaites et al. (2023) by shifting the analytical emphasis from predictive indicators of retention to the lived experiences of attrition, thereby contributing to the evidence base for developing targeted and contextually relevant retention strategies and supports.

The study findings underscore the importance of employee recognition in retaining PCWs. Recognition, one of Hollinger-Smith’s (2003) three Rs of retention, highlights the significant impact that managerial relationships play in retaining direct care workers. Recognition has been identified as one of the most cost-effective and easily implementable strategies. Creapeau et al.’s (2022) qualitative study involving PCWs, directors of nursing and RAC administrators across 59 RAC facilities, found that recognition of PCWs for performing a ‘good job’ was among the most effective strategies for promoting their retention. These insights illustrate the importance of recognition not only through financial incentives but also through formal acknowledgement of PCWs’ contribution, reinforcing their value to the organization and ultimately enhancing employee retention.

Our study highlights the importance of visible, supportive and approachable leadership in enhancing retention among PCWs. Within the published literature on effective employee retention strategies, the practice of visible and supportive leadership, particularly through initiatives such as Gemba walk, has consistently demonstrated value (Aij & Teunissen, 2017). Derived from the Japanese term meaning ‘the actual place’, Gemba involves structured leadership visibility and indirect support, where managers conduct regular walks through the shop floor with frequent stops to engage with employees and observe operations firsthand. This practice allows leaders to gain a deeper understanding of the challenges faced by their workforce (Aij & Teunissen, 2017). In the context of RAC, visible leaders who are regularly ‘walking the floor’, enables direct engagement with frontline staff and residents, providing first-hand insights into the realities of providing care and facilitating the identification of strategies to better support PCWs and improve retention outcomes, consistent with the findings of Aij and Teunissen (2017).

According to Montague et al. (2015), the challenges of attracting and retaining direct care workers in RAC requires urgent, cost-effective, and innovative policy and human resource reforms. As PCWs spend the most amount of time with residents compared to other workers, participants in this study described the importance of managers recognizing and valuing their deep knowledge of residents’ preferences and needs. The relational knowledge, developed through sustained, direct care, was considered central to both quality of care and employee satisfaction. By valuing PCWs’ expertise, organizations can foster a more respected, motivated and stable workforce, supporting the kind of structural change advocated by Montague et al. (2015).

In our study, continuing training and skill development emerged as another factor enhancing employee retention, with a particular emphasis on the need for specialist training, especially in palliative and dementia care. These findings align with the recommendations of the Royal Commission into Aged Care Quality and Safety (RCACQS) (2021), which emphasized the importance of continual professional development for the aged care workforce. Given the expansive and relatively complex responsibilities of PCWs, including the requirement to be responsive to the evolving needs of residents (Aged Care Workforce Strategy Taskforce, 2018) along with the introduction of new technologies, a skills deficit persists (RCACQS, 2021). Although 66% of PCWs possess an entry-level vocational qualification (Certificate III or IV in Aged Care or Individual Support or higher) (AGDH, 2020), research indicates that many are inadequately prepared for the multifaceted demands of their roles (Booi et al., 2021). Emerging literature emphasizes the insufficiency of pre-employment training (Booi et al., 2021), a shortcoming that our findings further underscored, with participants expressing a heightened level of discomfort and uncertainty upon commencing their role. The findings reinforce the need to prioritize a comprehensive, structured onboarding process and continuing training programs to strengthen employee preparedness to enhance employee retention.

In the demanding and highly stressful environment of RAC, supporting the mental health and well-being of PCWs is essential. The role is characterized by fast-paced environments, heavy workloads and time constraints (RCACQS, 2021), which is further compounded by emotionally challenging situations such as caring for residents with deteriorating health and providing end-of-life care. These challenges increase the risk of compassion fatigue, emotional exhaustion and burnout among PCWs (RCACQS, 2021). In response to these risks, our findings suggest that measures such as counselling services, promoting self-care practices and implementing staff well-being programs would benefit PCWs and improve their retention in RAC.

Our finding regarding the importance of supporting the recovery of injured workers as a retention strategy has received little attention in existing literature. Given the labor-intensive nature of the role, predominantly held by females (Australian Government, 2020), it is unsurprising that lifting and transferring aged residents is identified as a physical safety concern (Chang et al., 2013). Furthermore, aged care support workers frequently suffer from musculoskeletal injuries due to these physical demands (Chang et al., 2013). Our findings highlight the need to support injured workers, including assisting them with navigating the workers’ compensation insurance system and facilitating a safe return to work process. Existing literature indicates that adherence to occupational health and safety recommendations appears to be closely linked to the prevailing workplace culture, further highlighting the importance of fostering a supportive environment (Amateau et al., 2023).

Strengthening the workforce through diverse skills-mix and adequate staffing levels, in the current study, was also identified as contributing to employee retention. Empirical evidence suggests that higher staffing levels not only enhance care quality (Castle et al., 2020) but also directly correlate with the retention of PCWs and improvement across quality indicators (Castle, 2021). These findings take on an added urgency in the context of Australia’s aging population, increasing demand for aged care services, and multidimensional workforce issues facing RAC operators including the persistent shortfalls in the PCW workforce. Without urgent and sustained investment in strengthening the aged care workforce as identified in this study, the sector risks undermining the very foundation of person-centered care. This study reinforces the need for a national retention strategy that prioritizes continuity of care through workforce stability, acknowledging that individualized, relationship-based care is not achievable without retaining the people who provide it.

Optimizing work schedules and managing workloads through flexible working arrangements emerged as contributing factors to PCW retention. Participants indicated that being able to select shifts to accommodate personal commitments is essential for maintaining work-life balance in a sector that operates around the clock to provide continuous care and support. Moreover, our findings corroborate existing literature on the benefits of short shifts and limiting shift lengths as useful retention supports (Amateau et al., 2023).

We identified that an inclusive, collaborative and respectful workplace culture is important in fostering PCW retention. Participants emphasized that an inclusive and collaborative working environment characterized by open, transparent communication, respectful dialogue, and collective problem solving contributed to greater workforce stability by cultivating a sense of respect, belonging and trust rather than marginalization. This aligns with prior research indicating that support from co-workers, supervisors and managers increases the likelihood of employee retention (Creapeau et al., 2022). Furthermore, these insights from our study support Riggs and Rantz’s (2001) assertion that nursing homes should be viewed not merely as organizations, but rather as communities of workers, wherein a supportive and inclusive culture is integral to workforce retention.

Since Stone and Dawson’s (2008) seminal work on Better Jobs Better Care, America’s largest coordinated effort, conducted across five states to improve recruitment and retention in the RAC workforce, the foundational principles for supporting and sustaining the direct care workforce have been well established. In the two decades since the initiative, research specific to Australia’s PCW workforce has remained relatively sparse (Dhakal et al., 2020; Howe et al., 2012; Radford et al., 2015), and, unlike America, large-scale, system-wide interventions have been notably absent.

While America’s aged care system is fragmented, market-driven, and varies significantly across states, Australia’s aged care system is nationally standardized and centrally regulated (Dyer et al., 2019). Notwithstanding these key structural and operational differences between the two aged care systems, Australia’s structural coherence favorably positions it for implementation of a nationally coordinated workforce retention strategy. However, despite this advantage, Australia continues to face workforce shortages, compounded by high employee turnover and low levels of retention, suggesting that regulation alone is insufficient without a significant financial investment.

Given the stark difference in turnover rates, estimated at 129% annually for Certified Nursing Assistants in America (Miller et al., 2023) and the relative policy inertia in Australia, it is clear that evidence alone does not drive reform. Australia’s aged care system must move beyond piecemeal solutions as identified by the Royal Commission into Aged Care Quality and Safety (RCACQS, 2021) and instead adopt a nationally coordinated, long-term strategy informed by the learnings from Better Jobs Better Care. Without such action, Australia’s aged care sector risks repeating the well-documented failures of the past (RCACQS, 2021), despite decades of clear evidence about what works.

The findings of this study are especially salient and timely, considering the Australian Government’s introduction of the new *Age Care Act 2024*, to commence on 1 November 2025 (Australian Government, 2024), marking the transition from a market-driven to a rights-based, person-centered care approach (RCACQS, 2021). A key attribute of rights-based, person-centered care is understanding the person and their lived experience (Wilberforce et al., 2017), which is achieved through continuity and consistency of care. However, this cannot be achieved without a stable and committed personal care workforce. These reforms are unfolding in parallel with an increasing shortfall in the PCW workforce due to difficulties in attracting and retaining PCWs, and in addition to a growing population of older people (Castle et al., 2007; Denny-Brown et al., 2020; RCACQS, 2021). This study offers unique and valuable insights into issues that are driving the largest segment of the aged care workforce to leave the role. Capturing the voices of former employees, an underrepresented group in employee retention research reveals the complex and often overlooked realities behind workforce attrition. These insights must inform future workforce policy if the aged care sector is to deliver on the key foundational components of the new *Aged Care Act 2024 *(Cth).

### Implications

The findings of this study illustrate strategies and supports to promote PCW retention, offering practical guidance for further consideration by RAC operators, policymakers, and researchers. With an increased reliance on the PCW workforce, alongside the growing demand for aged care services, there is a need to implement evidence-informed retention interventions aimed at retaining the PCW workforce and ensuring the continuity of safe, responsive and high-quality care for residents in RAC.

Importantly, this study draws on the perspectives of PCWs who have left the aged care sector, an often unique, underrepresented yet essential source of insight for informing retention strategies and supports. Our findings reveal specific gaps in current human resource practices and point to targeted areas for improvement, including leadership visibility and engagement, recognition of PCW contributions, enhanced onboarding and continual training, improving workforce capacity and flexibility, prioritizing employee well-being and injury management, and fostering an inclusive and respectful workplace culture. Redirecting efforts towards these areas may support the implementation of practical, evidence-informed retention interventions to enhance PCW retention.

Further research to explore and evaluate the implementation and longitudinal effects of these strategies and supports across diverse care settings, with particular attention to context-specific factors, such as organization size, ownership type, and location, is recommended. Building an evidence base around what works and what does not work, for whom, and under what conditions (Graham & McAleer, 2018) will be critical for refining suitable, sector-wide retention models.

### Study Strengths and Limitations

The rigorous, qualitative methodology employed in this study captured participants’ perspectives and experiences, providing a deep understanding of employee retention from individuals who have left the aged care sector. These insights provide valuable guidance for RAC operators, policymakers, and researchers in developing strategies and supports to promote the retention of PCWs. However, several limitations must be acknowledged. First, recruitment challenges were encountered, as social media was the sole method used, potentially excluding individuals with limited digital access or digital literacy. This may have introduced sample selection bias, limiting the transferability of the findings. While recruitment via social media and participant self-selection present limitations that may affect the transferability of the study’s findings, the study’s rigor, underpinned by reflexive thematic analysis, strengthens the validity of the insights. Additionally, as participants were self-selected to participate, important perspectives from former PCWs who were unaware of the study or chose not to participate may have been missed. However, the study’s sample was considered sufficient based on the principle of information power. Finally, participants may have provided socially desirable responses rather than fully disclosing their true experiences and views to the interviewer.

## Conclusion

Given the growing demand for aged care services, driven by an aging population and the heavy reliance on the PCW workforce to provide most of the care and support to residents in RAC, the sustainability of this workforce is critical. The findings from this qualitative study provide a valuable contribution to identifying strategies and supports to enhance the retention of PCWs in RAC. Specifically, the role of visible, empathetic leadership and continuing training programs emerged as instrumental in promoting PCW job satisfaction and commitment. Additionally, addressing gaps in human resource practices, such as a structured onboarding process, workload management, and workplace culture, can promote the retention of PCWs.

We recommend that RAC operators, policymakers, and researchers use the findings of this study to further develop and co-design strategies and supports that are tailored to the specific needs of PCWs and the organizational context to strengthen workforce retention. Additionally, we recommend that the retention strategies and supports be implemented and longitudinally evaluated across diverse RAC contexts to refine and adapt approaches to contextual nuances. Ultimately, addressing PCW retention through targeted, multi-level interventions is imperative to ensuring workforce sustainability and the provision of safe, high-quality person-centered care.

## Data Availability

Data may be available from the corresponding author upon request.*
